# Comparing Strategies to Prevent Stroke and Ischemic Heart Disease in the Tunisian Population: Markov Modeling Approach Using a Comprehensive Sensitivity Analysis Algorithm

**DOI:** 10.1155/2019/2123079

**Published:** 2019-01-29

**Authors:** Olfa Saidi, Martin O'Flaherty, Nada Zoghlami, Dhafer Malouche, Simon Capewell, Julia A. Critchley, Piotr Bandosz, Habiba Ben Romdhane, Maria Guzman Castillo

**Affiliations:** ^1^Cardiovascular Epidemiology and Prevention Research Laboratory, Faculty of Medicine of Tunis, University Tunis El Manar, Tunis, Tunisia; ^2^Department of Public Health and Policy, University of Liverpool, Liverpool, UK; ^3^National Institute of Statistics and Data Analysis Tunis, Tunis, Tunisia; ^4^Population Health Research Institute, St George's University of London, London, UK

## Abstract

**Background:**

Mathematical models offer the potential to analyze and compare the effectiveness of very different interventions to prevent future cardiovascular disease. We developed a comprehensive Markov model to assess the impact of three interventions to reduce ischemic heart diseases (IHD) and stroke deaths: (i) improved medical treatments in acute phase, (ii) secondary prevention by increasing the uptake of statins, (iii) primary prevention using health promotion to reduce dietary salt consumption.

**Methods:**

We developed and validated a Markov model for the Tunisian population aged 35–94 years old over a 20-year time horizon. We compared the impact of specific treatments for stroke, lifestyle, and primary prevention on both IHD and stroke deaths. We then undertook extensive sensitivity analyses using both a probabilistic multivariate approach and simple linear regression (metamodeling).

**Results:**

The model forecast a dramatic mortality rise, with 111,134 IHD and stroke deaths (95% CI 106567 to 115048) predicted in 2025 in Tunisia. The salt reduction offered the potentially most powerful preventive intervention that might reduce IHD and stroke deaths by 27% (−30240 [−30580 to −29900]) compared with 1% for medical strategies and 3% for secondary prevention. The metamodeling highlighted that the initial development of a minor stroke substantially increased the subsequent probability of a fatal stroke or IHD death.

**Conclusions:**

The primary prevention of cardiovascular disease via a reduction in dietary salt consumption appeared much more effective than secondary or tertiary prevention approaches. Our simple but comprehensive model offers a potentially attractive methodological approach that might now be extended and replicated in other contexts and populations.

## 1. Background

Cardiovascular diseases (CVDs) cause nearly one-third of all deaths worldwide. 80% of these deaths occur in low-income and middle-income countries. Ischemic heart diseases (IHD) and stroke account for the greatest proportion of CVDs [1–3].

The burden of IHD and stroke is considerable, and they are the first and second leading causes of death, respectively, worldwide [4, 5]. They accounted for 15.2 million deaths (15.0 million to 15.6 million) in 2015 [4]. According to the World Health Organization (WHO), there are 15 million people worldwide who suffer from stroke each year, among them, 5 million die and another 5 million are left permanently disabled, causing a heavy burden for the family and community.

The burden of stroke will increase significantly over the next 20 years, particularly in developing countries [6]. Thus, the analysis of the effectiveness of health promotion interventions is urgently required for appropriate planning to reduce this burden [7].

Traditional epidemiological study designs cannot address these issues; even clinical trials are usually restricted by inclusion and exclusion criteria and not necessarily generalizable to the entire population [8].

Mathematical modeling overcomes many of these limitations. It plays a crucial role in helping to guide the most effective and cost-effective ways to achieve the goals of health promotion, designed and validated to guide health policies and development strategies at several levels [9, 10].

Briefly, a model is a simplification of a real situation and can encompass a simple, descriptive tool up to systems of mathematical equations [11].

The application of mathematical models in medicine has proved useful and has become more frequent, especially for cardiovascular diseases (CVDs) [12–16] and assessing potential impacts of policies or interventions designed to alter disease trajectories in Tunisia [17, 18].

A commonly used technique is Markov modeling, an approach that models groups of individuals transitioning across specified pathways, informed by transition probabilities [18–20].

Due to the frequent uncertainty of the Markov model's inputs, sensitivity analyses to assess the robustness of the model results are strongly recommended by modeling guidelines. Such uncertainty analyses assess confidence in a chosen course of action and ascertain the value of collecting additional information to better inform the decision [21].

Our study aims (i) to describe a comprehensive Markov model based on both a probabilistic multivariate approach and simple linear regression metamodeling and (ii) using the model to evaluate the effects of increases in uptake of stroke treatments lifestyle changes and primary prevention among the Tunisian population aged 35–94 years old in 2025. We examined three interventions: (a) improved medical treatments in the acute phase, (b) secondary prevention of stroke by increasing the prescribing of statins, and (c) primary prevention aiming to reduce salt intake.

## 2. Methods

In this study, we describe the development of a Markov IHD and stroke model.

### 2.1. Definition of Markov Model

**Figure 1:**
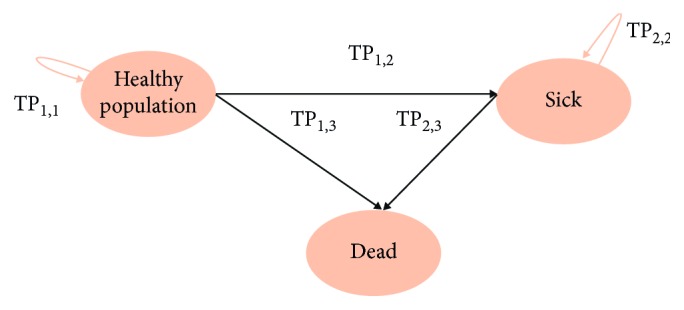
Markov diagram states and transition probabilities (each circle represents a Markov state and arrows indicate transition probabilities).

Markov models are a type mathematical model based on matrix algebra which describes the transitions a cohort of patients make among a number of mutually exclusive and exhaustive health states during a series of short intervals or cycles. In this model, a patient is always in one of the finite number of health states; events are modeled as transitions from one state to another and contribution of utility to overall prognosis depend on length of time spent in health states [19]. The components of a Markov model are shown below (Figure 1):

- States: the set of distinct health states under consideration in the model, together with the possible transitions between them.
- Cycle length: the length of time represented by a single stage (or cycle) in a Markov process: Markov models are developed to simulate both short-cycle and long-term processes (e.g., cardiovascular diseases).
- Transition probabilities: the matrix of probabilities of moving between health states from one stage to the next.

### 2.2. Process of Mathematical Modeling Based on Markov Approach

Before starting the data collection and the calculation, we first defined our model by specifying the different states that can be included based on the literature and expert opinions:

(1) $$\begin{array}{c} S=\left\{S_{1},S_{2},\ldots ,S_{3}\right\},\text{ set of states in the process}. \end{array}$$

Having specified the structure of the model in terms of the possible transitions between states, we defined the transition probabilities based on available data.

The transition probability (TP_*ij*_) is defined as a conditional probability (*P*_*t*_(*s*_*j*_/*s*_*i*_)) of making a transition (moving) from state *i* to state *j* during a single cycle (*t*). Additionally, transition probabilities are stratified by sex and age groups *a*_*g*_ ∈ {*c*_1_, *c*_2_,…, *c*_*g*_}, where *c*_1_, *c*_2_,…, *c*_*g*_ represents a set of age groups [19, 22].

One of the goals of the Markov model is to study the potential effects of some health promotion interventions. We modeled first a baseline scenario and then the intervention scenarios.

For the baseline scenario, we assumed no change will happen during the period “*T*” of study (20 years) in either the present uptake rates of medical therapies or population level uptakes of specific nutrients.

Based on the model structure in Figure 1, we assume that we will apply three interventions to study their impact on mortality in the future (over the 20 years: from *t* = year 1 to year 20).

We define a policy Π=(*I*_0_, *I*_1_, *I*_2_, *I*_3_) as a set of the health promotion interventions.

 
*I*_0_ refers the baseline scenario: we assumed no change will happen during 20 years.
 
*I*_1_: scenario aimed to improve medical treatments in acute phase.
 
*I*_2_: scenario for secondary prevention.
 
*I*_3_: scenario for primary prevention.

Starting by the baseline scenario, the process of the Markov model is based on the two first steps below:

**Step 1 I.**

Calculate probabilistic transition probabilities

Probabilistic sensitivity analysis aims to fully evaluate the combination of uncertainty in all model inputs (including transition probabilities) simultaneously on the robustness of model results.

The point estimates in the model can be replaced with probability distributions, where the mean of the distribution reflects the best estimate of the parameter.

In this step, each input parameter (TP_*ij*_) is assigned an appropriate statistical distribution, and a Monte Carlo simulation is run multiple times (e.g., 1000). The iteration is stopped when the difference of the outcomes is sufficiently small.

We assume for example that TPs follow the distribution defined by beta (*α*, *β*), where *α* probability distributions are defined on the interval [0, 1] parameterized by two positive shape parameters, denoted by *α* and *β*, that appear as exponents of the random variable and control the shape of the distribution [23].

**Step 2 II.**

Calculate the number of people in each state for the baseline scenario

The next step is to define the number of people in each state based on the TPs between states and the number of people in the starting state “healthy people.”

In this stage, we define the number of people in each state for the baseline scenario (healthy people (NHP), sick (NS), and deaths (ND)) for all gender and age groups from *t* = year 1 to year *T*, given by the following equations:

(2) $$\begin{array}{c} {\text{NHP}}_{t}={\text{NHP}}_{t-1}\times \left(1-\sum {\text{TP}}_{i,j}\right),\\ {\text{NS}}_{t}={\text{NS}}_{t-1}+\left({\text{NHP}}_{t-1}\times {\text{TP}}_{1,2}\right),\\ {\text{ND}}_{t}={\text{ND}}_{t-1}+\left({\text{NHP}}_{t-1}\times {\text{TP}}_{1,3}\right)+\left({\text{NS}}_{t-1}\times {\text{TP}}_{2,3}\right). \end{array}$$

Another indicator can be calculated based on this model, that is, life years gained (LY) defined by the following equation:

(3) $$\begin{array}{c} {\text{LY}}_{t}={\text{NHP}}_{t}-{\text{ND}}_{t}. \end{array}$$

The model is then run several times (e.g., 1000 simulations). For each simulation model, parameter values are randomly drawn from each of the distributions, and the expected model outcome is recorded. The 1000 simulations result in a distribution of expected model outcomes (e.g., deaths), which reflects the overall parameter uncertainty in the mode [24].

At the end of the simulation, the mean as well as the lower bound (LB) and upper bound (UB) of 95% confidence interval of the inputs and outputs will be calculated, which correspond to Steps I and II presented in Algorithm 1.

**Step 3 III.**

Calculate the number of people in each state for the interventions scenarios

For the interventions scenarios, the model required a base estimate of risk reduction in deaths and the age effect to calculate policy effectiveness (Π_*e*_^*Ii*^) of each intervention.

The policy effectiveness is defined by the following formula:

(4) $$\begin{array}{c} \overset{Ii}{\underset{e}{\prod }}=1-\left({\text{RR}}_{i}\times a_{e}\right), \end{array}$$

where RR_*i*_ is the risk reduction associated with the intervention *I*_*i*(*i*=1 : 3)_ obtained from previous randomized controlled trials and meta-analyses to estimate the reduction in age-specific deaths and *a*_*e*_ represents the age effects for each intervention risk reduction value. Π_*e*_^*Ii*^ is then used to scale the transition probabilities connected to death states.

In this stage, after recalculating the TPs, we define the number of people in each state (healthy people (NHP^*Ii*^), sick (NS^*Ii*^), and deaths (ND^*Ii*^) for all sex and age groups from *t* = year 1 to year *T* for the interventions scenarios *I*_*i*_, given by the following equations:

(5) $$\begin{array}{c} {\text{NHP}}_{t}^{li}={\text{NHP}}_{t-1}^{li}\times \left(1-{\text{TP}}_{1,2}-{\text{TP}}_{1,3}\times \overset{Ii}{\underset{e}{\prod }}\right),\\ {\text{NS}}_{t}^{li}={\text{NS}}_{t-1}^{li}+\left({\text{NHP}}_{t-1}^{li}\times {\text{TP}}_{1,2}\right),\\ {\text{ND}}_{t}^{li}={\text{ND}}_{t-1}^{li}+\left({\text{NHP}}_{t-1}^{li}\times {\text{TP}}_{1,3}\right)+\left({\text{NS}}_{t-1}^{li}\times {\text{TP}}_{2,3}\times \overset{Ii}{\underset{e}{\prod }}\right),\\ {\text{LY}}_{t}^{li}={\text{NHP}}_{t}^{li}-{\text{ND}}_{t}^{li}. \end{array}$$

**Step 4 IV.**

Calculate the final outputs (DPPs and LY)

Finally, we calculate the total number of CVD deaths (ischemic stroke and IHD deaths) that could be prevented or postponed (DPPs) and the life years gained (LY) under each specific scenario (equations (6) and (7)), compared to the baseline scenario for all sex and age groups from *t* = year 1 to year *T* for the interventions scenarios *I*_*i*_.

(6) $$\begin{array}{c} {\text{DPP}}_{s_{t}}^{li}={\text{ND}}_{t}^{li}-{\text{ND}}_{t}^{l0}, \end{array}$$

(7) $$\begin{array}{c} {\text{LY}}_{g_{t}}^{li}={\text{LY}}_{t}^{li}-{\text{LY}}_{t}^{l0}. \end{array}$$

**Step 5 V.**

Linear regression metamodeling

Most current modeling studies are limited to the first four stages and ignore this important step of metamodeling to analyze which model inputs are most influential in affecting the results. The goal of metamodeling was thus to increase the transparency of decision-making analytic models and better communicate their results.

**Figure 2:**
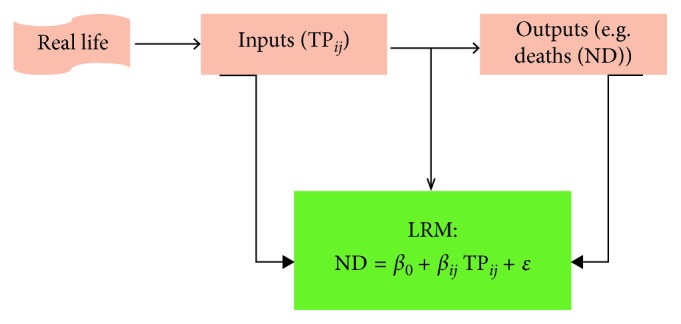
Simple linear regression metamodel (LRM) to summarize the relationship between model inputs and outputs.

This step is based on the application of a simple linear regression metamodel (LRM) for the optimal policy (Figure 2) [25].

The original motivation for metamodeling was to define a simpler mathematical relationship between model outputs and inputs than the actual model.

The LRM is defined by the following formula:

(8) $$\begin{array}{c} \text{ND}=\beta_{0}+\beta_{i,j}{\text{TP}}_{i,j}+\varepsilon , \end{array}$$

where ND is the number of deaths (output of the model); *β*_0_ (the intercept) is the expected outcome when all parameters are set to zero; TP_*i*,*j*_, the transition probabilities (inputs); *β*_*i*,*j*_, the other coefficients, are interpreted relative to a 1-unit change in each parameter on the original scales; and *ε* is the residual term.

Furthermore, the absolute value of the coefficients of the parameters “*β*_*i*,*j*_” can be used to rank parameters by their importance: the higher the coefficient is, the more the variable is important and relevant.

The algorithm below summarizes the process with three states that could be generalized to *n* states depending on the context to study (Algorithm 1).

### 2.3. Case Study

The algorithm introduced above has been implemented using R (Version R.3.2.0.) software and applied on Tunisian data. The source codes are available from the authors upon request.

Our model predicts both IHD and stroke deaths in 2025 among the Tunisian population aged 35–94 years old, both men and women, and compares the impact of specific treatments for stroke, lifestyle changes, and primary prevention.

### 2.4. Model Structure

Data were integrated and analyzed using a closed cohort model based on a Markov approach, with transition probabilities starting from population free of ischemic stroke and moving to health states reflecting the natural history of ischemic stroke [26, 27]. Therefore, the starting states are defined by the size of the population and the number of strokes occurring within this population. The number of persons moving from the starting states to the stroke and death states is estimated by the transition probabilities (Table 9 in the appendix in the supplementary materials).

There are two absorbing states: IHD and stroke deaths and non-IHD and stroke deaths as competing risks for mortality. IHD and stroke have several risk factors in common; increased salt intake is associated with hypertension which is one of the major risk factors for both stroke and IHD. Therefore, any policy aimed at decreasing population level's intake of salt will reduce the risk of both diseases [28].

**Figure 3:**
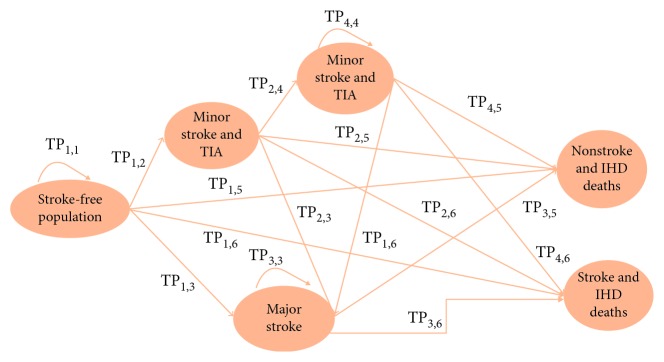
Stroke model structure. TIA: transient ischemic attack; IHD: ischemic heart diseases.

Potential overlaps between the healthy, minor, major stroke, and deaths are managed by calculating the conditional probabilities of membership. A key element of the model is the calculation of the transition probabilities (TPs), particularly those related to stroke and IHD mortality (Figure 3). The TP calculations are detailed in Table 10 in the Appendix in the supplementary materials.

### 2.5. Baseline Scenario

In the baseline scenario, we assumed there would be no change over the 20-year model period in the present uptake rates of acute phase medical treatments (thrombolysis, aspirin, and stroke unit) or treatments for secondary prevention following stroke (aspirin, statin, anticoagulants, blood pressure control, and smoking cessation) or treatment and policies for primary prevention (blood pressure control, glucose control, lipid lowering, salt uptake, and smoking cessation).

### 2.6. Prevention Scenarios

In this paper we evaluated the following three scenarios:

1. *I*_1_: the first scenario aimed to improve medical treatments in the acute phase: increasing thrombolysis prescribing from 0% to 50% and hospitalization in stroke units from 10% to 100% in Tunisia.
2. *I*_2_: the second scenario aimed to act on medical treatments after a stroke: increasing the prescribing of statins from 11% to 100% (secondary prevention).
3. *I*_3_: the third scenario aimed to reduce the consumption of salt by 30%, from 14 grams to 9 grams per day (primary prevention).

Total policy refers to the combined effects of all the three previous strategies: acute treatment + secondary prevention + primary prevention.

### 2.7. Modeling Policy Effectiveness and Its Impact in Mortality

The model applies the relative risk reduction (RRR), as mentioned in the algorithm above, quantified for each intervention scenario in previous randomized controlled trials and meta-analyses based on international studies (data are detailed in the Appendix in the supplementary materials (Table 8)).

### 2.8. Data Sources

Published and unpublished data were identified by extensive searches, complemented with specifically designed surveys. Data items included (i) number of stroke patients (minor and major), (ii) uptake of specific medical and surgical treatments, (iii) population data in the initial study year (2005) and (iv) mortality data (after one year (data in 2006) and after 5 years (data in 2010)). Data sources are detailed in Appendixin the supplementary materials.

### 2.9. Model Outputs

The outputs of this model are the prediction of stroke and IHD deaths prevented or postponed (DPPs) and the life years gained (LY) among the Tunisian population aged 35–94 years old starting from 2005 over a twenty-year time period(to 2025) with and without possible interventions to reduce this mortality.

We modeled all the intervention scenarios to calculate the total number of CVD deaths (ischemic stroke and IHD deaths) that may be prevented or postponed and the LY for each specific scenario compared to the baseline scenario.

## 3. Results

### 3.1. Baseline Scenario

In the baseline scenario, the model forecast 111140 [95% CI 106570 to 115050] IHD and ischemic stroke deaths for people aged 35–94 years between 2005 and 2025, including 68890 [95% CI 65730 to 72350] among men and 42250 [95% CI 38840 to 44600] among women.

The model estimated that the acute stroke treatment and secondary prevention following stroke would, respectively, prevent 230 [95% CI 200 to 260] deaths due to stroke and IHD and 1920 [95% CI 1830 to 2000] in 2025, whilst primary prevention could prevent 20330 [95% CI 20050 to 20610] cumulative deaths due to stroke and IHD in 2025.

In terms of life years, 150 [95% CI 130 to 180] and 2390 [95% CI 2300 to 2490] would be gained in 2025 by acute stroke treatment and secondary prevention, respectively, whereas for primary prevention (blood pressure control, glucose control, lipid lowering, and smoking cessation), 14590 [95% CI 14350 to 14820] life years would be gained in 2025 (Table 1).

### 3.2. Scenario Projections

#### 3.2.1. Improvement on Acute Stroke Treatment

If we adopt a strategy to increase thrombolysis uptake from 0% to 50% and stroke units use from 10% to 100%, 600 [95% CI 550 to 650] fewer deaths could be achieved by 2025 (Table 2).

**Figure 4:**
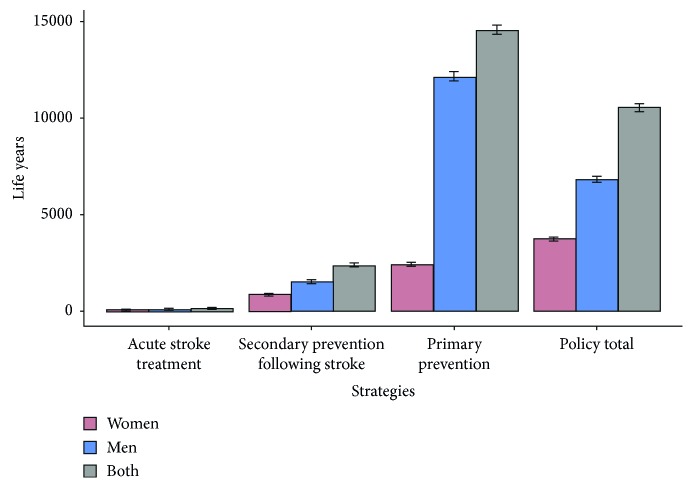
Life years estimations by incorporating strategies by gender in 2025.

In terms of life years, 370 [95% CI 330 to 410] would be gained in 2025 by increasing thrombolysis uptake (Figure 4).

#### 3.2.2. Improvement on Secondary Prevention

If the uptake of statins for secondary prevention following stroke could be increased from 11% to 100%, 3300 [95% CI 3190 to 3410] fewer deaths could be avoided by 2025 (Table 2).

In terms of life years, 4120 [95% CI 4000 to 4250] would be gained in 2025 (Figure 4).

#### 3.2.3. Food Policies

If the Tunisian government implemented a strategy recommended by the WHO to reduce the daily consumption of salt by 30 % (from 14 g/day to 9 g/day), 30240 [95% CI 29900 to 30580] deaths could be avoided by this scenario in 2025 (Table 2). This results in 20630 [95% CI 20350 to 20910] life year gain by 2025 (Figure 4).

### 3.3. Linear Regression Metamodeling of the Optimal Policy

The table below shows the results of linear regressing parameters on the stroke and IHD deaths prevented or postponed (DPPs) by the salt reduction intervention. The *R* ^2^ is 0.8999, suggesting that 89. 99% of the variance in the model outcomes is explained by our model (Table 3).

**Figure 5:**
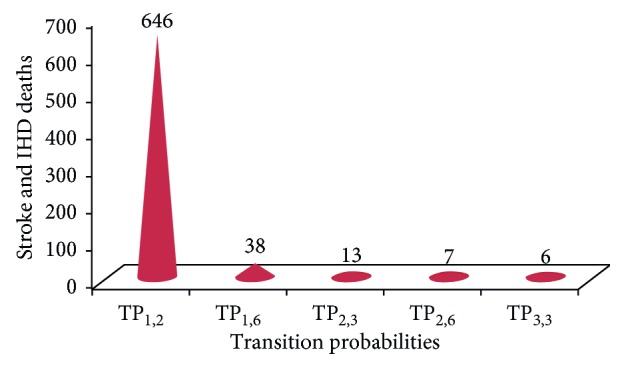
Five first important parameters in the model.

Figure 5 shows the five first important parameters based on the absolute value of the coefficients of the parameters. In our case study, the uncertainty of the probability of minor stroke in the first year has the greatest impact on the stroke and IHD deaths estimates associated with salt reduction, followed by the probability of the stroke-free population to die from stroke and IHD causes in the year 1 and the probability of recurrent stroke in ischemic stroke patients after one year. Although the main objective of the metaregression is to identify the most important parameters of the model, it could also serve to give a rough idea of the size of the effect of each parameter. We know that for each unit increase in the independent variable, our outcome should increase by ${\hat{I}}^{2}$ units. For example, the probability for the stroke-free population to have first stroke in the year 1 has an absolute coefficient of 646.35; this means that for each 10% risk increase in this probability, strokes and IHD will increase by 65 IHD and stroke deaths. However, this should be interpreted with caution since the meta-analysis regression model assumes a linear relationship between outcomes and inputs which is not the case in a Markov model (Figure 5).

## 4. Discussion

In this study, we have developed a simple but comprehensive Markov model and used it to identify key factors that predict mortality from stroke and IHD in Tunisia in the future, as well as the potential impacts of some medical and health policies.

Different mathematical models have been highly used in medical decision making [19, 29–32], but the technique of metamodeling is less developed in medicine. It has been used to identify the importance of variables that can justify the best medical decisions among pregnant women with deep vein thrombosis [33]. Additionally, linear regression metamodels have also been used in some epidemiological studies [25, 34].

This analysis is the first modeling study of stroke and IHD mortality in Tunisia, based on a rigorous and comprehensive modeling approach consisting of three stages:

- Apply the Markov model in medical and strategic decision making.
- Provide a probabilistic sensitivity analysis.
- Use a linear regression metamodeling for the optimal policy.

This model has several strengths. First it requires multiple epidemiological national data on ischemic stroke and demographics. In general, the data used in the model were of good quality. However, some assumptions were necessary to fill gaps in the missing information. We made transparent assumptions with clear justification (see Appendix in the supplementary materials).

Another advantage characterizing our approach and differentiating it from the univariate traditional method of deterministic sensitivity analysis is that it allows varying all the parameters of the model simultaneously and thus exploring the whole parameter space. The use of only one-way sensitivity analyses is limited compared with multiway analyses [35].

Furthermore, to increase the transparency of decision-making analytic models, we have used metamodeling in this study by applying a simpler mathematical relationship between model outputs and inputs to analyze which are the most influential inputs on the output.

Our metamodeling approach summarizes the results of the model studied in a transparent way and reveals its important characteristics.

The intercept of the regression result is the expected outcome when all parameters are set to zero. The other coefficients are interpreted relative to a 1-unit change in each parameter on the original scales. For example, in our case, changing the risk of the first minor stroke in the first year from the actual value to 1 increases the number of stroke and IHD deaths by 646 deaths. In addition, the regression coefficients describe the relative importance of the uncertainty in each parameter. And the use of the linear metamodeling regression method can overcome the limitations of other traditional statistical methods [36].

In addition, models often ignore the interaction effect between the input parameters of a model when defining the results. However, in our study, the use of conditional probabilities allows us to take into account interactions in our algorithm.

We also analyzed, in parallel of the deaths prevented or postponed (DPPs), the life years gained according to the different scenarios. Thus, our approach allows interpreting two key indicators in epidemiology via a rigorous and comprehensive mathematical algorithm.

Nevertheless this modeling approach has also some limitations; in fact, a major limitation of our work is that the model is a closed cohort, and demographic changes were not considered.

Linear regression is the metamodeling approach most widely used by many researchers for its simplicity and ease of use. Thus, our linear approximation of all input parameters can be considered as a limit, as state-transition models are nonlinear in general. In all metamodeling approaches, the loss of some information is always inevitable [25].

Another limitation of our study is not to consider in the model the costs of the strategies.

In terms of public health, the present modeling study focuses on the future impact of ischemic stroke treatment scenarios and population-level policy interventions on ischemic stroke and IHD deaths and life years gained in Tunisia. The model forecast a dramatic rise in the total cumulative number of IHD and Stroke deaths: by 2025, this number was estimated to reach more than 100,000 deaths.

Secondly, the model shows that the rise of thrombolytic treatment and hospitalization in intensive care units of stroke increased statin use for secondary prevention pale in comparison with the salt reduction impact on future deaths (1%, 3% vs 27% deaths prevented by 2025).

The benefits of thrombolytic treatment among patients with acute ischemic stroke are still matter of debate: thrombolytic treatment increases short-term mortality and symptomatic or fatal intracranial haemorrhage but decreases longer term death or dependence [25]. Many studies proved that stroke units have significant benefit to patient outcomes in terms of reducing mortality, morbidity, and improving functional independence. Stroke unit care was also cost effective [37–39].

The substantial effect of salt reduction intervention found in our study is consistent with the literature [40, 41]. High salt intake is associated with significantly increased risks of stroke and total cardiovascular disease ranging from a 14% to 51% increase, depending on salt intake and populations [42–44].

Furthermore, our results are consistent with a modeling study in Tunisia using a different approach. Different strategies for salt reduction were associated with substantial lowering of CVD mortality and were cost saving apart from health promotion [45] and consistent with the Rose hypothesis that the population level strategies are more appropriate in terms of less medicalization [46].

## 5. Conclusions

The approach presented here is attractive since it is based on a simple comprehensive algorithm to present the results of sensitivity analysis from the Markov model using linear regression metamodeling. This approach can reveal important characteristics of Markov decision process including the base-case results, relative parameter importance, interaction, and sensitivity analyses.

Thus, we recommend using this algorithm for Markov decision process; it can be the object of creation of a complete modeling package in R software and can be extended to other contexts and populations.

In terms of public health, we forecast that absolute numbers of IHD and stroke deaths will increase dramatically in Tunisia over 2005–2025. This Large increase in stroke and IHD mortality in Tunisia needs many actions not only in acute stroke treatment such as implementing more basic and organized stroke units but also in population level primary prevention such as salt reduction in order to manage and treat acute strokes and to alleviate the global burden of these diseases.

Our study highlights the powerful impact of salt reduction on deaths from stroke and IHD. Furthermore, the reducing dietary salt intake across the population appears an effective way of reducing heart disease events and saving substantial costs. This result matches with that of the mathematical model. Indeed our metamodeling highlights that the probability of the first minor stroke among the healthy population has the greatest impact on the stroke and IHD death estimations, which confirms the importance of primary prevention. Prevention is thus the best strategy to fight against stroke and IHD deaths.

## Figures and Tables

**Algorithm 1:**
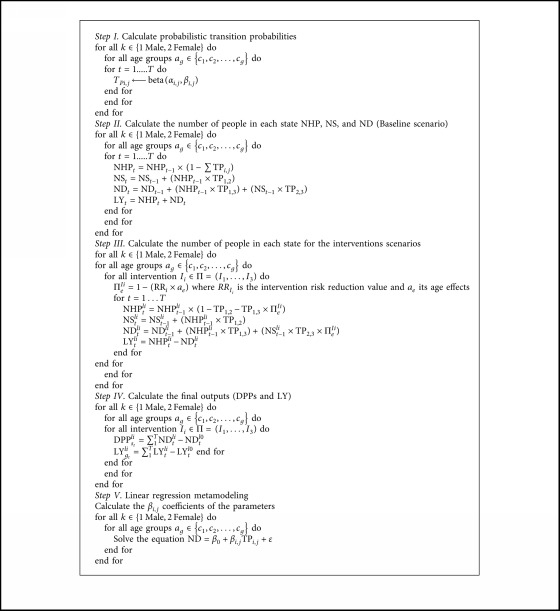
Comprehensive algorithm of the Markov model and of sensitivity analysis.

**Table 1:** Life years and deaths due to stroke and IHD estimations in 2025 keeping the same practices of 2005 by gender.

	Life years [95% CI]	Stroke and IHD deaths [95% CI]
*Men*		
Acute stroke treatment	80 [70 to 100]	−140 [−170 to −120]
Secondary prevention following stroke	1500 [1420 to 1580]	−1170 [−1240 to −1110]
Primary prevention	12180 [11960 to 12400]	−16760 [−17020 to −16510]
Policy total^*∗*^	6830 [6700 to 6990]	−8530 [−8710 to −8340]
*Women*		
Acute stroke treatment	60 [50 to 80]	−80 [−100 to −70]
Secondary prevention following stroke	860 [800 to 920]	−720 [−770 to −670]
Primary prevention	2410 [2310 to 2510]	−3570 [−3690 to −3450]
Policy total^*∗*^	3730 [3610 to 3850]	−4860 [−5000 to −4720]
*Both*		
Acute stroke treatment	150 [130 to 1804]	−230 [−260 to −200]
Secondary prevention following stroke	2390 [2300 to 2490]	−1920 [−2000 to −1830]
Primary prevention	14590 [14350 to 14820]	−20330 [−20610 to −20050]
Policy total^*∗*^	10560 [10360 to 10770]	−13380 [−13610 to −13160]

^*∗*^Total policy refers to the combined effects of all the three previous strategies: acute treatment + secondary prevention + primary prevention.

**Table 2:** Life years and deaths due to stroke and IHD by incorporating strategies in 2025 by gender.

	Stroke and IHD deaths [95% CI]
*Men*	
Acute stroke treatment	−350 [−390 to −310]
Secondary prevention following stroke	−2060 [−2150 to −1970]
Primary prevention	−24500 [−24810 to −24200]
Policy total	−23940 [−24240 to −23640]
*Women*	
Acute stroke treatment	−220 [−250 to −190]
Secondary prevention following stroke	−1240 [−1310 to −1170]
Primary prevention	−10630 [−10830 to −10430]
Policy total	−17050 [−17300 to −16800]
*Both*	
Acute stroke treatment	−600 [−650 to −550]
Secondary prevention following stroke	−3300 [−3410 to −3190]
Primary prevention	−30240 [−30580 to −29900]
Policy total^*∗*^	−40990 [−41390 to −40600]

^*∗*^Total policy refers to the combined effects of all the three previous strategies: acute treatment + secondary prevention + primary prevention.

**Table 3:** Regression coefficients from metamodeling on the stroke and IHD deaths by the salt reduction intervention.

Parameters	Dependent variable (*Y*): stroke and IHD deaths
Intercept	1337.86
TP_1,2_: probability for the stroke-free population to have first stroke in the year 1	646.35
TP_1,3_: probability for the stroke-free population to have first major stroke in the year 1	6.18
TP_1,6_: probability for the stroke-free population to die from stroke and IHD causes in the year 1	38.49
TP_1,1_: probability for population free of stroke	2.45
TP_2,3_: probability of recurrent stroke in ischemic stroke patients after 1 year	13.05
TP_2,6_: probability for the minor stroke patients to die from stroke and IHD causes in the year 1	6.67
TP_2,4_: probability for first minor stroke (1st year) to minor stroke subsequent years	0.45
TP_3,3_: probability of recurrent stroke in ischemic stroke patients after 5 years	−6.49
TP_4,6_: probability for the stroke patients to die from stroke and IHD deaths causes 1 year after first admission	−1.72
TP_4,4_: probability for minor stroke subsequent years	−2.96
TP_3,6_: probability for the major stroke patients to die from stroke and IHD causes	−0.10
Observations	1000
*R* ^2^	0.8999

## Data Availability

Data used are presented in Appendix in the supplementary materials.

## Supplementary Materials

Supplementary Materials The supplementary materials submitted contain sources of data and all data used in the model.
