# Supplementary material for: Comparing Strategies to Prevent Stroke and Ischemic Heart Disease in the Tunisian Population: Markov Modeling Approach Using a Comprehensive Sensitivity Analysis Algorithm
Source: Comput Math Methods Med. 2019 Jan 29;2019:2123079. doi: 10.1155/2019/2123079 (PMC6374861; doi:10.1155/2019/2123079)
Supplement: Supplementary Materials — The supplementary materials submitted contain sources of data and all data used in the model. [file 2123079.f1.docx]

**Comparing strategies to prevent stroke and ischemic heart disease in the Tunisian population: Markov modeling approach using a comprehensive sensitivity analysis algorithm**

O. Saidi *et al.*

# *Technical Appendix*

Contents

[1 Overview 2](#_Toc507365438)

[2 Data sources and assumptions 2](#_Toc507365439)

[2.1 POPULATION 2](#_Toc507365440)

[2.2 TRANSITION PROBABILITIES 2](#_Toc507365441)

[2.2.1 Ischemic stroke patients in 2005 2](#_Toc507365442)

[Assumptions 3](#_Toc507365443)

[2.2.2 Total deaths 3](#_Toc507365444)

[2.2.3 CVD deaths 4](#_Toc507365445)

[2.2.4 ACUTE STROKE TREATMENT 6](#_Toc507365446)

[2.2.5 TREATMENT FOR SECONDARY PREVENTION FOLLOWING STROKE 6](#_Toc507365447)

[Assumptions 7](#_Toc507365448)

[2.2.6 TREATMENT FOR PRIMARY PREVENTION 8](#_Toc507365449)

[2.2.7 Policy effectiveness and its impact in mortality 8](#_Toc507365450)

[3 Transitions probabilities 10](#_Toc507365451)

***List of tables***

[Table 1 : Tunisian population aged 35 years old and over by gender-Tunisia 2005 2](#_Toc507365291)

[Table 2: ischemic stroke patient numbers in Tunisia (2005) 3](#_Toc507365292)

[Table 3 : Total deaths in Tunisia 2006, 2010 4](#_Toc507365293)

[Table 4 : CVD deaths in Tunisia -2006,2010 5](#_Toc507365294)

[Table 5 : Acute stroke treatment in Tunisia -2005 6](#_Toc507365295)

[Table 6 : Treatment for secondary prevention following stroke-2005 7](#_Toc507365296)

[Table 7 : Treatment for primary prevention-2005 8](#_Toc507365297)

[Table 8: Relative risk reductions for CVD deaths from previous studies for intervention scenarios 9](#_Toc507365298)

[Table 9: Transitions probabilities 10](#_Toc507365299)

[Table 10: Markov stroke states 10](#_Toc507365300)

# Overview

The Stroke Model was developed using R Software Version R.3.2.0. This model integrates specific epidemiological and demographic Tunisian data to analyze the impact of specific treatment of stroke, life style and primary prevention on both IHD and stroke deaths in 2025 (20 years from the start year study's(2005)) among Tunisian population aged 35-94 years old. In this document, we provide details about calculations of transitions probabilities, the data sources, estimates and assumptions used in the model.

# Data sources and assumptions

## POPULATION

We obtained the population data from National Institute of Statistic-Tunisia* (Table 1).

Table 1 : Tunisian population aged 35 years old and over by gender-Tunisia 2005

|  | Male | Female | Total |
| --- | --- | --- | --- |
| 35-44 | 671200 | 705700 | 1376900 |
| 45-54 | 521600 | 516300 | 1037900 |
| 55-64 | 282400 | 298700 | 581100 |
| 65-74 | 226200 | 224500 | 450700 |
| 75-84 | 83933 | 79133 | 163066 |
| 85-94 | 27978 | 26378 | 54356 |
| >=95 | 1813311 | 1850711 | 3664022 |
| Total | **671200** | **705700** | **1376900** |

Source: National Institute of Statistic-Tunisia

* The National Institute of statistics represents a central organ in the national statistics system: The institute conducts regular surveys and census (every 10 years) and publishes specific reports on demographic indicators: Annual report and population projection up to 2032. (Online publication: [www.ins.nat.tn](http://www.ins.nat.tn) )

## TRANSITION PROBABILITIES

### Ischemic stroke patients in 2005

We obtained the total number of stroke admissions in 2005 from the National survey 2003-2004 conducted in cardiology services for hospitals, the study included all individuals without limitations of age or sex admitted in hospital cardiology services. The total number patients was 48665 patients.

Assumptions

- We have the total number of stroke admissions in 2003-2004 in public hospitals.
- We inflated it by 5% to estimate the total stroke admissions in public and private hospitals in 2003-2004 (Expert opinions).
- We have the annual incidence of Tunisian National Institute of Neurology (which represent 18% of our data in 2003-2004).
- We assumed that ischemic stroke represents 83%(Diagnosis of stroke subtype in a multicenter Cohort study (Neurology/ original )2001-2005).

|  |  | TIA | IS patients (EX TIA) | Total Ischemic stroke | % TIA / total IS |
| --- | --- | --- | --- | --- | --- |
| Male | **35-44** | 3 | 73 | 76 | 4,26 |
|  | **45-54** | 14 | 162 | 176 | 7,97 |
|  | **55-64** | 24 | 231 | 255 | 9,31 |
|  | **65-74** | 47 | 417 | 464 | 10,23 |
|  | **75-84** | 33 | 226 | 260 | 12,87 |
|  | **85-94** | 9 | 76 | 85 | 10,17 |
|  | **>=95** | 1 | 1 | 2 | 47,90 |
|  | **Total** | 132 | 1186 | 1318 | 9,88 |
| Female | **35-44** | 6 | 60 | 66 | 9,76 |
|  | **45-54** | 13 | 140 | 153 | 8,48 |
|  | **55-64** | 32 | 214 | 246 | 13,16 |
|  | **65-74** | 35 | 330 | 364 | 9,48 |
|  | **75-84** | 30 | 215 | 245 | 12,33 |
|  | **85-94** | 6 | 39 | 45 | 14,32 |
|  | **>=95** | 0 | 4 | 4 | 0,00 |
|  | **Total** | 123 | 1000 | 1122 | 11,01 |
| Total | **35-44** | 10 | 133 | 142 | 6,82 |
|  | **45-54** | 27 | 302 | 329 | 8,21 |
|  | **55-64** | 56 | 445 | 501 | 11,20 |
|  | **65-74** | 82 | 746 | 828 | 9,90 |
|  | **75-84** | 64 | 441 | 505 | 12,61 |
|  | **85-94** | 15 | 115 | 130 | 11,61 |
|  | **>=95** | 1 | 5 | 6 | 18,69 |
|  | **Total** | **255** | **2186** | **2441** | **10,40** |

**We have the total number of ischemic stroke admissions in 2005 (Table 2).**

**Table 2: ischemic stroke patient numbers in Tunisia (2005)**

### Total deaths

We obtained the total deaths in 2006 (after 1 year from the study year) and 2010 (after 5 years from the study year) from National Institute of Statistic-Tunisia (Table 3).

**Table 3 : Total deaths in Tunisia 2006, 2010**

|  | Total deaths 2006 | | | Total deaths 2010 | | |
| --- | --- | --- | --- | --- | --- | --- |
|  | **Male** | **Female** | **Total** | **Male** | **Female** | **Total** |
| 35-44 | 1512 | 934 | 2446 | 1414 | 889 | 2303 |
| 45-54 | 2723 | 1395 | 4118 | 2558 | 1399 | 3957 |
| 55-64 | 3573 | 2033 | 5606 | 3963 | 2070 | 6033 |
| 65-74 | 7131 | 4791 | 11922 | 6573 | 4415 | 10988 |
| 75-84 | 8478 | 6747 | 15225 | 9154 | 7480 | 16635 |
| 85-94 | 4369 | 3865 | 8234 | 4803 | 4299 | 9102 |
| >=95 | 743 | 818 | 1561 | 817 | 909 | 1727 |
| Total | **28529** | **20583** | **49112** | **29282** | **21461** | **50745** |

Source: National Institute of Statistic-Tunisia

### CVD deaths

We obtained the total deaths in 2006 (after 1 year from the study year) and 2010 (after 5 years from the study year) from National Public Health Institute and National Institute of Statistic-Tunisia (Table 4).

**Assumptions**

- Since 2001: The National Public Health Institute recorded causes of death according to the ICD10, using the STIX software:
- All deaths are registered,
- Only 50% of primary causes of death are recorded. However, in large cities the percentage is almost 80%.
- Extrapolation: Using National Institute of Statistics for mortality data, we applied the % of the recorded causes of death to the total number of deaths registered for 2006 and 2010.

Table 4 : CVD deaths in Tunisia -2006,2010

|  |  | 2006 | 2010 |
| --- | --- | --- | --- |
| Male | **35-44** | 181 | 225 |
|  | **45-54** | 550 | 551 |
|  | **55-64** | 1086 | 1089 |
|  | **65-74** | 2253 | 1963 |
|  | **75-84** | 2789 | 3182 |
|  | **85-94** | 1419 | 1630 |
|  | **>=95** | 259 | 277 |
|  | **Total** | **8537** | **8918** |
| Female | **35-44** | 113 | 178 |
|  | **45-54** | 332 | 320 |
|  | **55-64** | 481 | 559 |
|  | **65-74** | 1781 | 1359 |
|  | **75-84** | 2685 | 3246 |
|  | **85-94** | 1650 | 2199 |
|  | **>=95** | 346 | 438 |
|  | **Total** | **7388** | **8299** |
| Total | **35-44** | 294 | 403 |
|  | **45-54** | 882 | 872 |
|  | **55-64** | 1566 | 1648 |
|  | **65-74** | 4034 | 3323 |
|  | **75-84** | 5474 | 6428 |
|  | **85-94** | 3069 | 3829 |
|  | **>=95** | 605 | 715 |
|  | **Total** | **15925** | **17217** |

### ACUTE STROKE TREATMENT

We obtained the acute stroke treatment data from the Hospital survey 2002-2009 conducted in the outpatient of internal medicine yard in Marsa university hospital. Medical records of patients admitted between 2002 and 2009 for stroke were reviewed (Table 5).

Table 5 : Acute stroke treatment in Tunisia -2005

|  |  | Aspirin (%) | Thrombolysis (%) | Stroke unit (%) |
| --- | --- | --- | --- | --- |
| Male | **35-44** | 75,0 | 0 | 0.121 |
|  | **45-54** | 95,8 | 0 | 0.101 |
|  | **55-64** | 96,7 | 0 | 0.103 |
|  | **65-74** | 100,0 | 0 | 0.085 |
|  | **75-84** | 96,9 | 0 | 0.072 |
|  | **85-94** | 90,0 | 0 | 0.072 |
|  | **Total** | 84,8 | 0 | 0.094 |
| Female | **35-44** | 100,0 | 0 | 0.103 |
|  | **45-54** | 100,0 | 0 | 0.094 |
|  | **55-64** | 78,6 | 0 | 0.095 |
|  | **65-74** | 95,8 | 0 | 0.072 |
|  | **75-84** | 100,0 | 0 | 0.057 |
|  | **85-94** | 100,0 | 0 | 0.092 |
|  | **Total** | 94,7 | 0 | 0.083 |

### TREATMENT FOR SECONDARY PREVENTION FOLLOWING STROKE

We obtained the treatment for secondary prevention following stroke data from the PREMISE I (2002) and PREMISE II (2010 ) surveys:

- **PREMISE I (2002):** the survey of secondary prevention of myocardial infarction. Conducted in all patients followed in the outpatient cardiology. Internal medicine and neurology of the university hospitals in the district of Tunis. The study consists of patients who consulted during the period between October 2002 and February 2003 and fulfilling the inclusion criteria. Included in the survey. patients (both sexes): the diagnosis is confirmed cardiovascular disease and / or cerebro-vascular defined by one or more of the following conditions: myocardial infarction. stable or unstable angina. stroke. transient ischemic attack; who received angioplasty. Carotid endarterectomy or cerebrovascular surgery.
- **PREMISE II (2010):** The survey of secondary prevention was conducted during the period between February 2010 and June 2010 in 600 patients followed in the outpatient cardiology of two university hospitals in the district of Tunis. Inclusion criteria: patients (both sexes) with confirmed diagnosis of cardiovascular disease and / or cerebrovascular disease defined by one or more of the following conditions: myocardial infarction, angina, stroke, CABG or PTCA, carotid endarterectomy or cerebrovascular surgery, hypertension, diabetes, dyslipidemia:
- >= 21 years old
- receiving treatment uptake
- With a duration of the disease more than one month and less than three years.

Assumptions

- We calculated an average between 2002 and 2010 to estimate the % of aspirin, statin, smoking cessation and BP control in 2005 (Table 6).

Table 6 : Treatment for secondary prevention following stroke-2005

|  |  | Aspirin | Statins | Smoking cessation | BP Control | Warfarin |
| --- | --- | --- | --- | --- | --- | --- |
| Male | **35-44** | 80,0 | 10,0 | 45,0 | 50,0 | 0 |
|  | **45-54** | 91,7 | 22,2 | 70,0 | 50,0 | 0 |
|  | **55-64** | 91,1 | 19,6 | 35,0 | 25,0 | 0 |
|  | **65-74** | 79,9 | 17,3 | 30,6 | 50,0 | 0 |
|  | **75-84** | 70,5 | 18,2 | 30,6 | 50,0 | 0 |
|  | **85-94** | 92,9 | 18,2 | 55,6 | 50,0 | 0 |
|  | **Total** | **86,2** | **17,4** | **38,3** | **40,0** | **0** |
| Female | **35-44** | 100,0 | 20,6 | 66,7 | 50,0 | 0 |
|  | **45-54** | 79,2 | 20,6 | 66,7 | 50,0 | 0 |
|  | **55-64** | 84,9 | 20,6 | 75,0 | 37,5 | 0 |
|  | **65-74** | 82,2 | 7,9 | 60,0 | 37,5 | 0 |
|  | **75-84** | 83,9 | 7,1 | 45,0 | 37,5 | 0 |
|  | **85-94** | 87,5 | 25,0 | 45,0 | 37,5 | 0 |
|  | **Total** | **84,9** | **11,1** | **59,5** | **33,3** | **0** |

### TREATMENT FOR PRIMARY PREVENTION

We obtained the treatment for primary prevention data from Tunisian National Survey- TAHINA- 2005 (Tables 7): The survey was cross-sectional from April to September 2005 and the target population was all 35-70 year old of both genders. With a total of 8007 subjects. It was based on a nationally representative stratified two-stage cluster sample of households according to the seven administrative regions of Tunisia.

Table 7 : Treatment for primary prevention-2005

|  |  | BP Control | Lipid lowering | Glucose control | Smoking cessation | Warfarin |
| --- | --- | --- | --- | --- | --- | --- |
| Male | **35-44** | 31,6 | 1,0 | 0.54 | 39,6 | 0 |
|  | **45-54** | 36,4 | 2,4 | 0.39 | 43,2 | 0 |
|  | **55-64** | 17,5 | 4,0 | 0.54 | 50,8 | 0 |
|  | **65-74** | 25,4 | 3,3 | 0.54 | 48,7 | 0 |
|  | **75-84** | 25,4 | 3,3 | 0.54 | 48,7 | 0 |
|  | **85-94** | 25,4 | 3,3 | 0.54 | 48,7 | 0 |
|  | **Total** | 26,5 | 2,2 | 0.49 | 44,2 | 0 |
| Female | **35-44** | 35,6 | 0,7 | 0.69 | 0,4 | 0 |
|  | **45-54** | 23,9 | 2,0 | 0.42 | 0,7 | 0 |
|  | **55-64** | 20,2 | 5,5 | 0.45 | 0,4 | 0 |
|  | **65-74** | 20,8 | 4,3 | 0.49 | 0,2 | 0 |
|  | **75-84** | 20,8 | 4,3 | 0.49 | 0,2 | 0 |
|  | **85-94** | 20,8 | 4,3 | 0.49 | 0,2 | 0 |
|  | **Total** | **22,7** | **2,4** | **0.46** | **0,5** | **0** |

### Policy effectiveness and its impact in mortality

The model applies the relative risk reduction (RRR) quantified for each intervention scenario in previous randomized controlled trials and meta-analyses based on overseas studies (Table 8).

Table 8: Relative risk reductions for CVD deaths from previous studies for intervention scenarios

| **Interventions** | **Relative risk reduction (RRR)** | **Description** |
| --- | --- | --- |
| **Acute phase** | | |
| Stroke Unit | 6,1 % (95 % CI:0,0009–11) | RRR for stroke death or dependency [1] |
| Thrombolysis Treatment | 11 % (95 % CI: 5–16) | RRR for stroke death or dependency if applied within 4.5 h [2] |
| Aspirin Treatment | 2,6 % (95 % CI: 0.4–4) | RRR for stroke death or dependency if treatment is 160–300 mg once daily, started within 48 h of onset[3] |
| **Secondary Prevention** | | |
| Aspirin Treatment | 3 % (95 % CI: 6–19) | RRR for vascular events (stroke or IHD death) if treatment is at any dose above 30 mg daily [4] |
| Statin Treatment | 12 % (95 % CI: (-1)–21) | RRR for recurrent stroke if LDL reduces by 1 mmol/L [5] |
| Warfarin Treatment | 61 % (95 % CI: 37–75) | RRR for recurrent stroke or systemic embolism among stroke patients with Transient Ischemic Attack or minor stroke due to atrial fibrillation when treated with anticougulation [6] |
| BP Control | 34 % (95 % CI: 21–44) | RRR for stroke based on BP reduction of 4–25 mmHG systolic or 3–13 Hg diastolic [7] |
| Smoking Cessation | 48 % (95 % CI: 29–57) | RRR for stroke death or dependency [8] |
| **Primary prevention** | | |
| BP Control | 46 % (95 % CI: 35–55) | RRR based on BP reduction 5 mmHg. This reduces the risk of stroke by an estimated 34 % and ischemic heart disease by 21 % from any pre-treatment level [9] |
| HbA1C Control | 7 % (95 % CI: 4–19) | RRR for stroke based on 0,9 % HbA1C reduction [10] |
| Warfarin Treatment | 64 % (95 % CI: 49–74) | RRR for stroke based on a meta-analysis with twenty-nine trials, adjusted-dose warfarin reduced stroke by 64%[11] |
| Salt Reduction | 17 % (95 % CI:6–43) | RRR for stroke by 5gr change in daily salt intake [12] |
| Transfat Reduction | 12 % (95 % CI:5.5–18.5) | RRR by replacing 1 % of energy from trans-fat with unsaturated fats for coronary heart disease [13] We assumed half effect for stroke |
| Saturated Fat Reduction | 13 % (95 % CI: 1–6) | RRR by replacing 5 % of energy from saturated fat with Polyunsaturated fats (PUFAs) for coronary heart disease [14] We assumed half effect for stroke |
| Fruit and Vegetables Intake | 4 % (95 % CI:3–8) | RRR for stroke by change in 1 unit of fruit and vegetables [15] |
| Smoking Prevalence Reduction | 1.9 % (95 % CI:1.5–2.3) | RRR by change in 1 % prevalence of smoking [16] |

# Transitions probabilities

The table below shows the transitions probabilities.

Table 9: Transitions probabilities

| TP | From | To | Description |
| --- | --- | --- | --- |
| TP_1,1_ | HP | HP | Population to population stroke free |
| TP_1,2_ | HP | MiS1y | Population to first minor stroke (1st year) |
| TP_1,3_ | HP | MaS | Population to first major stroke |
| TP_1,5_ | HP | Non Stroke and IHD deaths | Population to Non Stroke and IHD deaths |
| TP_1,6_ | HP | Stroke and IHD deaths | Population to Stroke and IHD deaths |
| TP_2,3_ | MiS1y | MaS | First minor stroke (1st year) to first major stroke |
| TP_2,6_ | MiS1y | Stroke and IHD deaths | First minor stroke (1st year) to Stroke and IHD deaths |
| TP_2,5_ | MiS1y | Non Stroke and IHD deaths | First minor stroke (1st year) to non Stroke and IHD deaths |
| TP_2,4_ | MiS1y | MiS | First minor stroke (1st year) to minor stroke subsequent years |
| TP_1,6_ | MiS | Stroke and IHD deaths | Minor stroke subsequent years to first major stroke |
| TP_4,6_ | MiS | Stroke and IHD deaths | Minor stroke subsequent years to Stroke and IHD deaths |
| TP_4,5_ | MiS | Non Stroke and IHD deaths | Minor stroke subsequent years to non Stroke and IHD deaths |
| TP_4,4_ | MiS | MiS | Minor stroke subsequent years to minor stroke subsequent years |
| TP_3,6_ | Mas | Stroke and IHD deaths | First major stroke to Stroke and IHD deaths |
| TP_3,5_ | Mas | Non Stroke and IHD deaths | First major stroke to non Stroke and IHD deaths |
| TP_3,3_ | Mas | MaS | First major stroke to major stroke |

The table 10 provides the Markov stroke states.

Table 10: Markov stroke states

| State (*m*) | Description | Number of people in each state |
| --- | --- | --- |
| HP | Healthy population (stroke free) |  |
| MiS1y | First minor stroke (1st year) | - MiS1y_t_=HP_t-1_× TP_1,2_ |
| MaS | Major stroke | - MaS_t_=[HP_t-1_× TP_1,3_] + [MiS1y_t-1_× TP_2,3_] + [MiS_t-1_× TP_1,6_]+ [MaS_t-1_× TP_3,3_] |
| MiS | Minor stroke subsequent years | MiS_t_= [MiS1y_t-1_× (1-(TP_2,3_+ TP_2,6_+ TP_2,5_)] +[ MiS_t-1_× (1-( TP_1,6_+ TP_4,6_+ TP_4,5_))] |
| SIHD | Stroke and IHD deaths* | SIHD_t_= [HP_t-1_× TP_1,6_]+ [MiS1y_t-1_× TP_2,6_]+[ MiS_t-1_× TP_4,6_]+[ MaS_t-1_× TP_3,6_]+ SIHD_t-1_ |
| NSIHD | Non Stroke and IHD deaths | NSIHD_t_=[ HP_t-1_× TP_1,5_] + [MiS1y_t-1_× TP_2,5_]+[ MiS_t-1_× TP_4,5_]+[ MaS_t-1_× TP_3,5_]+ NSIHD_t-1_ |

** Ischemic heart disease and stroke deaths were classified according to the International Classification of Diseases 10^th^ Revision*

**References:**

1. Stroke Unit Trialists C. Organised inpatient (stroke unit) care for stroke. Cochrane Database Syst Rev. 2007;17(4):CD000197. doi:10.1002/14651858.CD000197.pub2.
2. Wardlaw JM, Murray V, Berge E, Del Zoppo GJ. Thrombolysis for acute ischemic stroke. Cochrane Database Sys Rev. 2009;4:CD000213. doi:10.1002/14651858.CD000213.pub2.
3. Sandercock PA, Counsell C, Gubitz GJ, Tseng MC. Antiplatelet therapy for acute ischemic stroke. Cochrane Database Sys Rev. 2008;3:CD000029. doi:10.1002/14651858.CD000029.pub2.
4. Algra A, van Gijn J. Cumulative meta-analysis of aspirin efficacy after cerebral ischemia of arterial origin. J Neurol Neurosurg Psychiatry. 1999;66(2):255.
5. Amarenco P, Labreuche J. Lipid management in the prevention of stroke: review and updated meta-analysis of statins for stroke prevention. Lancet Neurology. 2009;8(5):453–63. doi:10.1016/S1474-4422(09)70058-4.
6. Saxena R, Koudstaal P. Anticoagulants versus antiplatelet therapy for preventing stroke in patients with nonrheumatic atrial fibrillation and a history of stroke or transient ischemic attack. Cochrane Database Systematic Rev. 2004;4:CD000187. doi:10.1002/14651858.CD000187.pub2.
7. Zhang H, Thijs L, Staessen JA. Blood pressure lowering for primary and secondary prevention of stroke. Hypertension. 2006;48(2):187–95. doi:10.1161/01.HYP.0000231939.40959.60.
8. Hankey GJ. Ischaemic stroke–prevention is better than cure. J R Coll Physicians Edinb. 2010;40(1):56–63. doi:10.4997/JRCPE.2010.111.
9. Law M, Wald N, Morris J. Lowering blood pressure to prevent myocardial infarction and stroke: a new preventive strategy. Health Technol Assess. 2003;7(31):1–94.
10. Ray KK, Seshasai SR, Wijesuriya S, Sivakumaran R, Nethercott S, Preiss D, et al. Effect of intensive control of glucose on cardiovascular outcomes and death in patients with diabetes mellitus: a meta-analysis of randomised controlled trials. Lancet. 2009;373(9677):1765–72. doi:10.1016/S0140-6736(09)60697-8.
11. Hart RG, Pearce LA, Aguilar MI. Meta-analysis: antithrombotic therapy to prevent stroke in patients who have nonvalvular atrial fibrillation. Ann Intern Med. 2007;146(12):857–67.
12. Strazzullo P, D’Elia L, Kandala NB, Cappuccio FP. Salt intake, stroke, and cardiovascular disease: meta-analysis of prospective studies. BMJ. 2009;339:b4567. doi:10.1136/bmj.b4567.
13. Mozaffarian D, Clarke R. Quantitative effects on cardiovascular risk factors and coronary heart disease risk of replacing partially hydrogenated vegetable oils with other fats and oils. Eur J Clin Nutr. 2009;63 Suppl 2:S22–33. doi:10.1038/sj.ejcn.1602976.
14. Jakobsen MU, O’Reilly EJ, Heitmann BL, Pereira MA, Balter K, Fraser GE, et al. Major types of dietary fat and risk of coronary heart disease: a pooled analysis of 11 cohort studies. Am J Clin Nutr. 2009;89(5):1425–32. doi:10.3945/ajcn.2008.27124.
15. Dauchet L, Amouyel P, Dallongeville J. Fruit and vegetable consumption and risk of stroke: a meta-analysis of cohort studies. Neurology. 2005;65(8):1193–7. doi:10.1212/01.wnl.0000180600.09719.53.
16. Saidi O, Ben Mansour N, O’Flaherty M, et al. Analyzing Recent Coronary Heart Disease Mortality Trends in Tunisia between 1997 and 2009. [PLoS One](https://www.ncbi.nlm.nih.gov/pubmed/?term=Analyzing+Recent+Coronary+Heart+Disease+Mortality+Trends+in+Tunisia+between+1997+and+2009) 2013 May 3;8(5):e63202. DOI: 10.
